# Gab2 deficiency suppresses high-fat diet-induced obesity by reducing adipose tissue inflammation and increasing brown adipose function in mice

**DOI:** 10.1038/s41419-021-03519-9

**Published:** 2021-02-26

**Authors:** Xinhui Wang, Yinan Zhao, Dekun Zhou, Yingpu Tian, Gensheng Feng, Zhongxian Lu

**Affiliations:** 1 School of Pharmaceutical Sciences, State Key Laboratory of Cellular Stress Biology, Xiamen University, 361005 Xiamen, Fujian China; 2 Department of Pathology, Division of Biological Sciences, University of California at San Diego, La Jolla, CA 92093 USA

**Keywords:** Fat metabolism, Obesity

## Abstract

Obesity is caused by a long-term imbalance between energy intake and consumption and is regulated by multiple signals. This study investigated the effect of signaling scaffolding protein Gab2 on obesity and its relevant regulation mechanism. Gab2 knockout (KO) and wild-type (WT) mice were fed with a standard diet (SD) or high-fat diet (HFD) for 12 weeks. The results showed that the a high-fat diet-induced Gab2 expression in adipose tissues, but deletion of Gab2 attenuated weight gain and improved glucose tolerance in mice fed with a high-fat diet. White adipose tissue and systemic inflammations were reduced in HFD-fed Gab2 deficiency mice. Gab2 deficiency increased the expression of *Ucp1* and other thermogenic genes in brown adipose tissue. Furthermore, the regulation of Gab2 on the mature differentiation and function of adipocytes was investigated in vitro using primary or immortalized brown preadipocytes. The expression of brown fat-selective genes was found to be elevated in differentiated adipocytes without Gab2. The mechanism of Gab2 regulating Ucp1 expression in brown adipocytes involved with its downstream PI3K (p85)-Akt-FoxO1 signaling pathway. Our research suggests that deletion of Gab2 suppresses diet-induced obesity by multiple pathways and Gab2 may be a novel therapeutic target for the treatment of obesity and associated complications.

## Introduction

Obesity is a global public health problem and identified as serious risk factor for a variety of metabolic diseases^1–3^ and cancers^4^. Multiple strategies are adopted for the treatment of obesity, such as exercise, healthy diet, bariatric surgery, and pharmacotherapy^5^. However, obesity is still not able to be effectively prevented due to the complex signal regulation of obesity^1^. The hazards of overweight remind us to explore safe and effective therapies for obesity and associated diseases.

Obesity is characterized by an abnormal expansion of adipocytes and accumulation of white adipose tissue (WAT) and can be caused and intensified by numerous factors, including abnormalities in lipid and glucose metabolism, inflammation, and diseases^6^. Especially, inflammations in adipose tissue are associated with the development of obesity, insulin resistance, and other metabolic diseases^7^. Recently, brown adipose tissue (BAT) was found to play a novel role in obesity^8^. Activation of BAT improves metabolic health and resists high-fat diet-induced obesity by elevating energy expenditure in a mouse model^9–11^. The heat production function of BAT depends on the uncoupling protein-1 (Ucp1), a protein resided in the inner mitochondrial membrane and exclusively expressed in brown adipocytes^12^. The deficiency of Ucp1^+^ brown and beige adipocytes in mice increases susceptibility to obesity, insulin resistance, and hyperglycemia^13^. Activated BAT drains circulating carbohydrates and lipids to produce heat, so as to protect against hyperglycemia and hyperlipidemia^14,15^. BAT additionally participates in lipoprotein processing and reduces excess cholesterol by promoting cholesterol transformation to bile acids in the liver^16^. Furthermore, as an endocrine organ-like WAT, activated BAT secretes multiple cytokines, such as TNF-*α*, adiponectin, leptin, and fibroblast growth factor type 21 (FGF21), to regulate metabolic homeostasis^17–19^.

Grb2-associated binding protein 2 (Gab2) is a scaffolding protein in the cytoplasm that participates in the amplification and integration of signal transduction when cells are stimulated by growth factors, cytokines, and antigens^20,21^. Gab2 recruits various intracellular downstream effectors, including Ras, Src, p85, Shp2, Crk, PLC γ, Shc, and SHIP^22,23^ to affect cell migration, proliferation, differentiation, or polarization in different types of cells, including immune cells^24–27^. Extensive evidence confirms that Gab2 is involved in pulmonary fibrosis^24^, osteopetrosis^28^, cardiac disease^20^, Alzheimer’s disease^29^, and several types of cancers^22,30^. Our previous research indicates that deletion of Gab2 in mice resists lipid accumulation in hepatocytes and hepatic steatosis induced by high-fat diet (HFD) by integrating multiple signal pathways, suggesting that Gab2 may be a potential target for preventing and treating fat accumulation diseases, including NAFLD and obesity^31^.

By using a transgenic mouse, the present study discovered that deletion of Gab2 attenuated the fat accumulation and weight gain caused by HFD, improved glucose tolerance, reduced inflammation in white adipose tissue, and enhanced metabolic capacity of brown adipose tissue. These observations suggested that Gab2 may act as a novel key modulator to regulate obesity and associated complications.

## Results

### Deletion of Gab2 in mouse protects against high-fat diet (HFD)-induced obesity

**Fig. 1:**
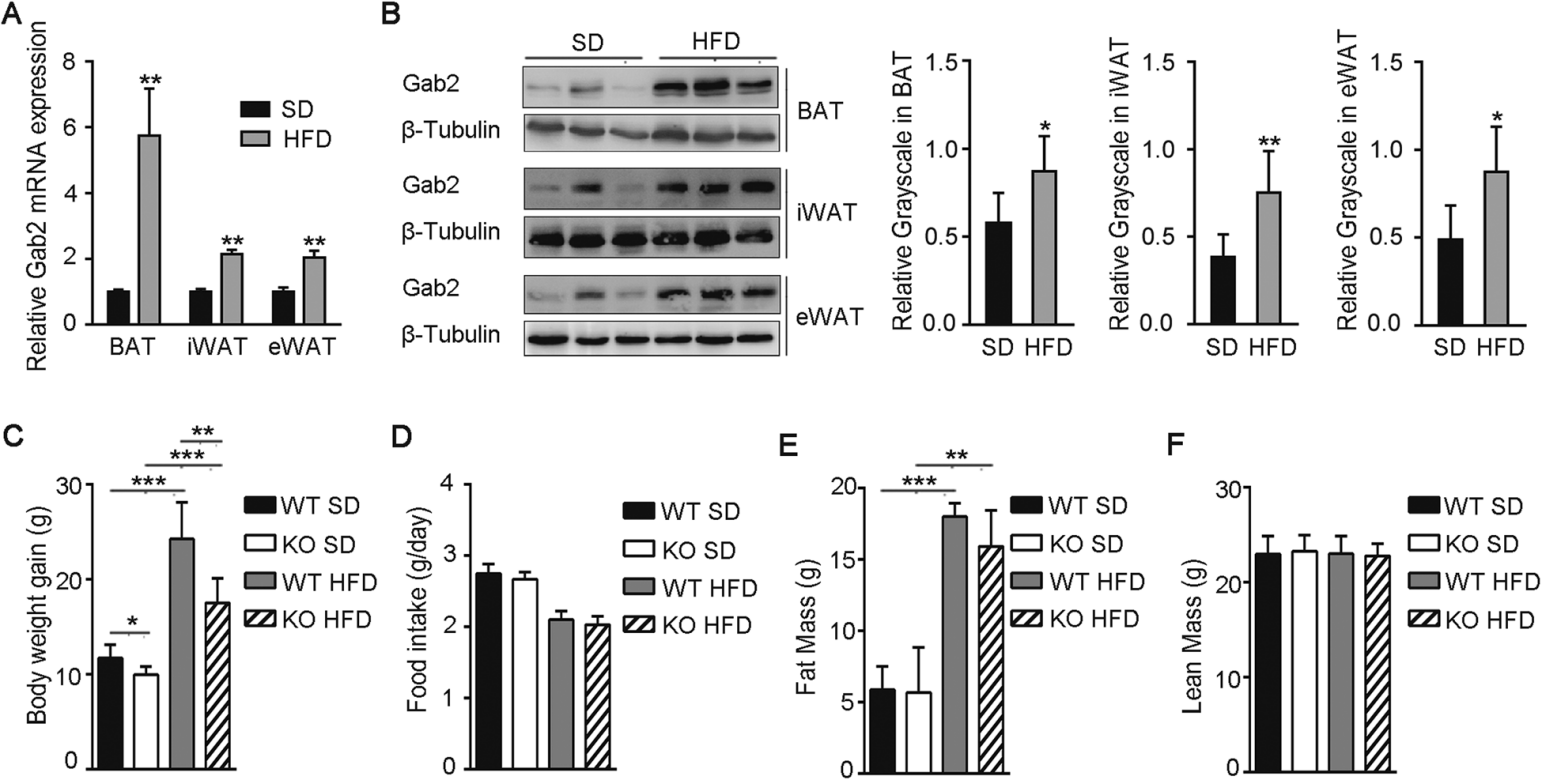
Gab2 is increased in adipose tissue in HFD-fed mice and deletion of Gab2 protects against HFD-induced obesity. **A** Relative mRNA level of Gab2 in brown adipose tissue (BAT), inguinal (iWAT), and epididymal (eWAT) white adipose tissue from mice fed with a standard diet (SD) or a high-fat diet (HFD) for 12 weeks detected by RT-qPCR. The results were normalized with S18 mRNA levels. **B** Left panel: representative blots of Gab2 protein expression in BAT (top), iWAT (middle), and eWAT (bottom) of mice described in **A** detected by immunoblotting analysis; Right panel: the relative grayscale values of Gab2 protein blots, β-Tubulin protein was measured as a loading control. **C**–**F** Bodyweight gain from weeks 6 to 18 (**C**), food intake (**D**), fat mass (**E**), and lean mass (**F**) of Gab2 KO and WT mice fed with SD and HFD diet for 12 weeks. The data are presented as mean ± SD from at least six mice in each group. Statistical difference is indicated: *P < 0.05; **P < 0.01; ***P < 0.001.

Male wild-type (WT) mice at 6 weeks of age were fed with HFD (60% fat) and grew significantly faster than mice fed with the standard diet (SD) (Supplemental Fig. 1A). After 12 weeks, the WT HFD-fed mice became obese and gained 38% more weight compared to the SD-fed controls (Supplemental Fig. 1A, B). To make clear the role of Gab2, its expression was firstly checked in BAT, inguinal WAT (iWAT), and epididymal WAT (eWAT). The results revealed that messenger RNA (mRNA) expression of Gab2 was significantly elevated in BAT (upregulated approximately 6 times), iWAT, and eWAT in WT mice fed with HFD compared to mice fed with a standard diet (SD) (Fig. 1A). Protein levels of Gab2 were increased around twofold in different adipose tissues (Fig. 1B). These observations indicated that Gab2 may affect the function of adipose tissue in obese mice.

To confirm this hypothesis, male Gab2 KO mice and their WT littermates at 6 weeks of age were fed with HFD. Deletion of Gab2 attenuated the HFD-induced weight gain in mice (Supplemental Fig. 1A). After 12 weeks of HFD feeding, Gab2 KO mice gained less weight (26%) compared to the SD-fed KO controls, which was significantly less than the weight gain of WT mice (38%, *P* < 0.01) (Fig. 1C and Supplemental Fig. 1A, B). Further research revealed that deletion of Gab2 did not affect the appetite of mice (Fig. 1D). In addition, the fat mass and lean mass of mice were analyzed. The results revealed that HFD increased the fat mass and had no influence on the lean mass, while deletion of Gab2 reduced the HFD-induced fat mass (Fig. 1E), without impact the lean mass (Fig. 1F), indicating that Gab2 has a regulation on fat accumulation.

**Fig. 2:**
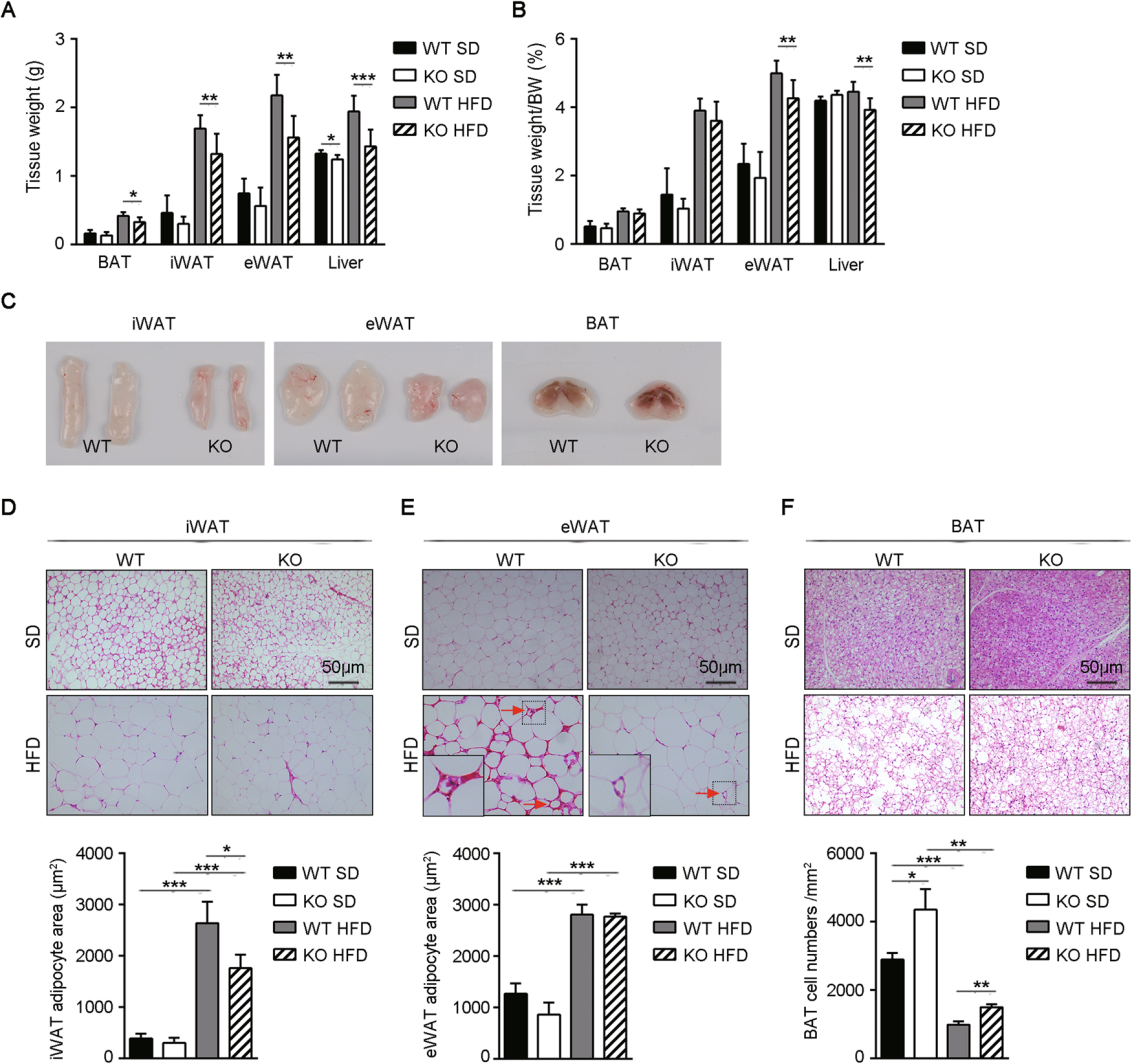
Deletion of Gab2 reduces the adipose tissue weight in mice fed with HFD. **A**, **B** Tissue weight (**A**) and tissue weight ratio to bodyweight (**B**) of Gab2 KO and WT mice fed with SD and HFD diet for 12 weeks. **C**–**F** Representative image (**C**), hematoxylin and eosin (H&E) staining and adipocyte area of iWAT (**D**), eWAT (**E**) and BAT (**F**) from Gab2 KO and WT mice fed with SD and HFD diet for 12 weeks, scale bar is 50 μm. The data are presented as mean ± SD from at least six mice in each group. Statistical difference is indicated: *P < 0.05; **P < 0.01; ***P < 0.001.

Consistent with this observation, deletion of Gab2 specifically reduced the adipose tissue in HFD-fed mice. The weight of tissue and the ratio of tissue to bodyweight of iWAT, eWAT, and BAT were prominently increased in the WT mice fed with HFD (Fig. 2A–C), while the HFD-induced hyperplasia of adipose tissues was significantly decreased in the Gab2 KO mice (Fig. 2A–C). Hyperplasia and hypertrophy of white adipocytes were clearly observed in iWAT and eWAT of HFD-fed mice, the adipocyte sizes of HFD-fed mice were significantly larger than SD-fed mice (Fig. 2D, E). However, the adipocyte size of iWAT from Gab2 KO mice was obviously smaller than that in WT mice after HFD feeding for 12 weeks (Fig. 2D and Supplemental Fig. 2A). In addition, there were obvious macrophage infiltration and “crown-like structures (CLS)” remodeling, characterized by a large number of macrophages surrounding dead adipocytes^32,33^, in eWAT of HFD-fed WT mice (Fig. 2E). Deletion of Gab2 remarkably decreased HFD-induced macrophage infiltration and CLS remodeling in eWAT, however, there was no significant difference in eWAT adipocyte size between WT and Gab2 KO mice (Fig. 2E and Supplemental Fig. 2B). Furthermore, large unilocular lipid droplets induced by HFD were observed in BAT of WT mice, while the Gab2 KO mice maintained smaller lipid droplets in brown adipocytes after feeding with HFD (Fig. 2F, top). Interestingly, Gab2 deficiency prominently increased the number of adipocytes in BAT of both SD and HFD-fed mice (Fig. 2F, bottom).

In addition, deletion of Gab2 also reduced the liver weight and hepatic lipid accumulation in HFD-fed mice (Fig. 2A, B and Supplemental Fig. 2C, D), which have been proved by our laboratory group in a NAFLD mouse model^31^.

All the results above indicated that Gab2 deficiency can diminish the development of obesity by affecting the number, size, and function of adipocytes.

### Gab2 ablation improves glucose tolerance and insulin sensitivity

**Fig. 3:**
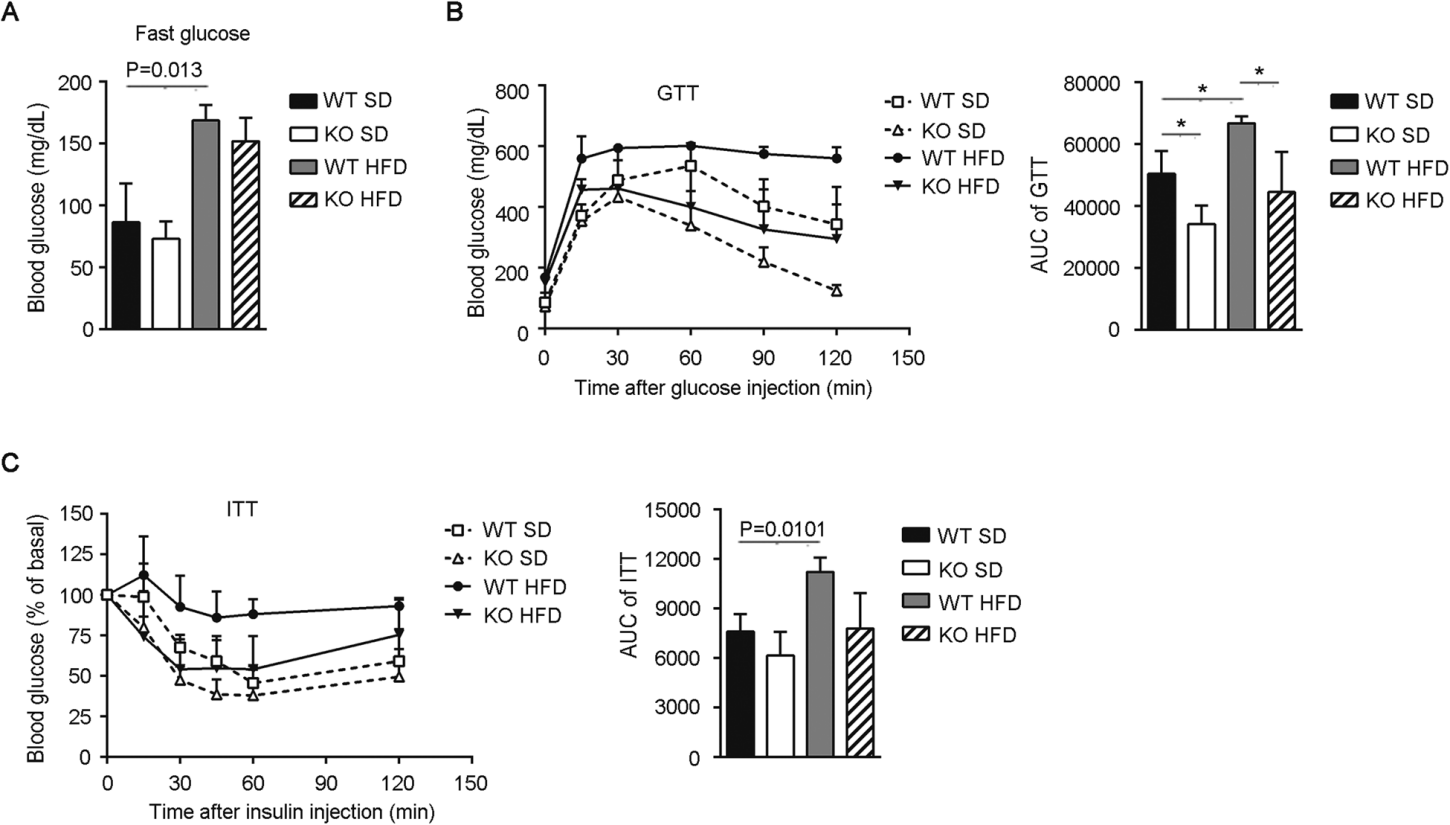
Deletion of Gab2 improves glucose tolerance and insulin sensitivity in HFD-fed mice. **A** Fasting blood glucose in Gab2 KO and WT mice fed with SD and HFD diet for 12 weeks. **B**, **C** Glucose tolerance test (GTT, **B**) and insulin tolerance test (ITT, **C**) performed in Gab2 KO and WT mice fed with SD and HFD diet for 12 weeks. Left panel, the curve of blood glucose concentration; Right panel, quantification of the area under the curve (AUC). The data are presented as mean ± SD from at least 6 mice in each group. Statistical difference is indicated: *P < 0.05; **P < 0.01.

The deposition of adipose tissue and adipocyte inflammation increases the risk of insulin resistance, which in turn exacerbates obesity and metabolic diseases^34^. Here, the influence of Gab2 on glucose homeostasis was evaluated in HFD-fed mice. First, the fasting blood glucose of mice fasted overnight was measured. The result showed that HFD raised the fasting blood glucose in mice after 12 weeks of feeding, but deletion of Gab2 had no obvious influence on the fasting blood glucose in both SD and HFD feeding groups (Fig. 3A). Furthermore, an oral glucose tolerance test (GTT) was performed. The HFD-fed mice presented higher level blood glucose concentrations during 120 min compared with the SD-fed mice. Deletion of Gab2 significantly reduced the blood glucose levels and improved glucose tolerance, and the area under the curve (AUC) was obviously less than that in HFD-fed WT mice (Fig. 3B, *P* < 0.05). Interestingly, Gab2 deficiency also improved glucose tolerance in SD-fed mice when compared with WT littermates (Fig. 3B, *P* < 0.05). Similarly, Gab2 KO mice possessed improved insulin sensitivity, in spite of no statistical difference was observed in the insulin tolerance test (ITT) assay (Fig. 3C). Our result demonstrated that deletion of Gab2 could improve glucose tolerance and insulin sensitivity in mice fed with HFD.

### Gab2 deficiency attenuates adipose tissue inflammation in HFD-fed mice

**Fig. 4:**
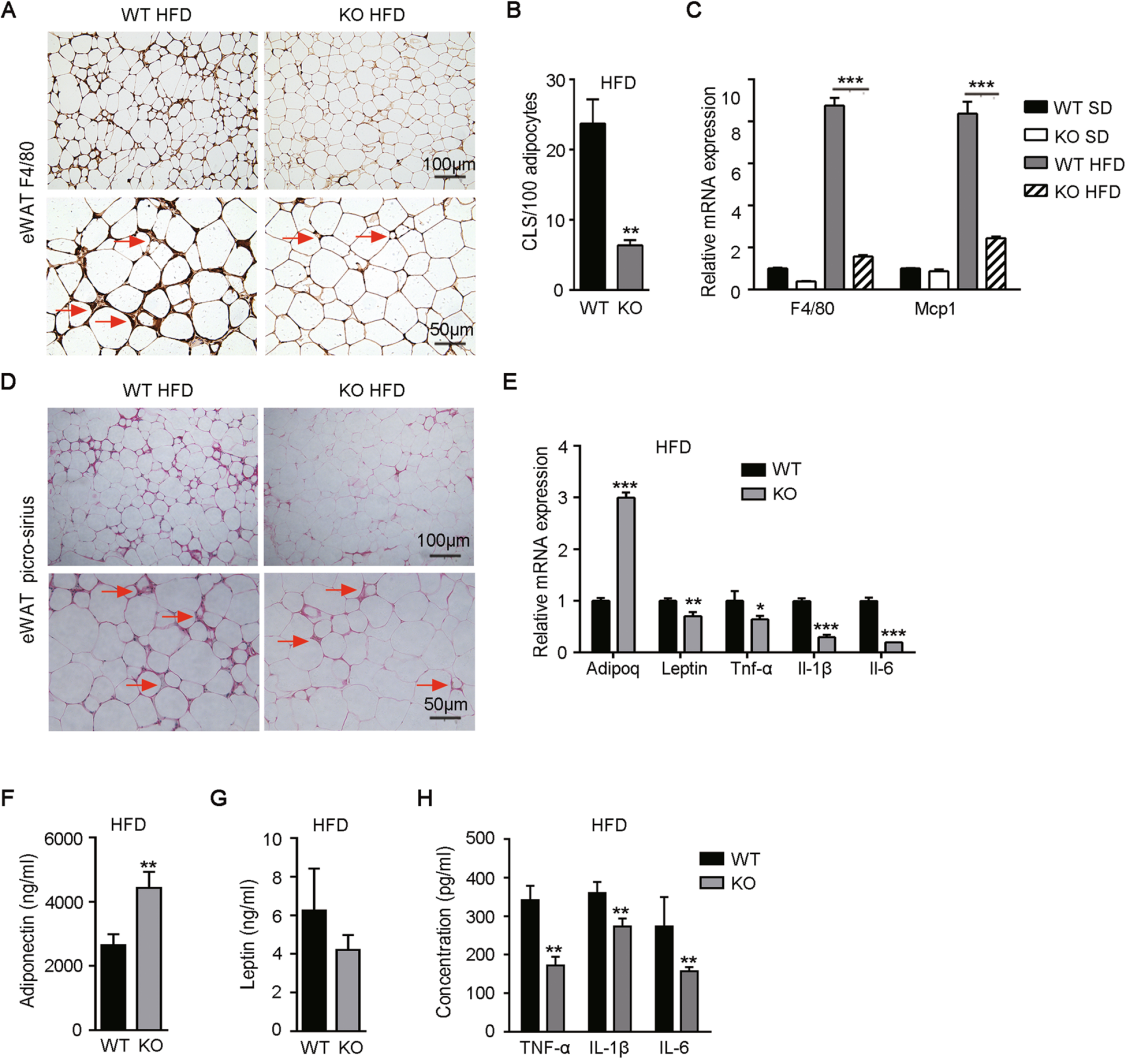
Gab2 deficiency reduces macrophage infiltration and inflammation in adipose tissue in HFD-fed mice. **A** Representative immunohistochemistry (IHC) staining images of F4/80 protein in eWAT from Gab2 KO and WT mice fed with HFD diet for 12 weeks. Crown-like structures are indicated by the red arrows. Scale bars are 100 μm (top) and 50 μm (bottom). **B** Quantification of crown-like structure (CLS) in eWAT from Gab2 KO and WT mice fed with HFD diet for 12 weeks. **C** The mRNA level of *F4/80* and *Mcp-1* in eWAT from Gab2 KO and WT mice fed with SD or HFD diet for 12 weeks were detected by RT-qPCR. The results were normalized with S18 mRNA levels. **D** Representative picro-sirius staining images of collagen fibrils in eWAT from Gab2 KO and WT mice fed with HFD diet for 12 weeks. Collagen fibrils are indicated by the red arrows. Scale bars are 100 μm (top) and 50 μm (bottom). **E** The mRNA level of *adiponectin* (*Adipoq*), *leptin*, and inflammation cytokines (*Tnf-α*, *Il-1β*, *Il-6*) in eWAT from Gab2 KO and WT mice fed with HFD diet for 12 weeks detected by RT-qPCR. The results were normalized with *S18* mRNA levels. **F**–**H** The concentration of adiponectin (**F**), leptin (**G**), and inflammation cytokines (TNF-α, IL-1β, IL-6, H) in blood plasma samples analyzed by ELISA assay. The data are presented as mean ± SD from at least 6 mice in each group. Statistical difference is indicated: *P < 0.05; **P < 0.01; ***P < 0.001.

Adipose tissue inflammation is associated with the development of obesity and insulin resistance. Immunohistochemical (IHC) staining for F4/80, a specific marker of macrophage^35^, was performed to confirm the inflammation in eWAT. The results revealed that there was a high density of crown-like structures (CLS) and abundant macrophages infiltration in eWAT from mice fed with HFD (Fig. 4A, red arrow indicated), while deletion of Gab2 significantly decreased HFD-induced macrophages infiltration and the formation of CLS (Fig. 4A, B). The drastically elevated mRNA expressions of *F4/80* and *Mcp-1* (another macrophage marker^36^) in eWAT from WT mice fed with HFD were significantly reduced in Gab2 KO mice (Fig. 4C). Adipose tissue inflammation and increasing adipose remodeling eventually result in the formation of fibrosis^37^. In our research, serious fibrosis was also observed in eWAT with picro-sirius staining in HFD-fed WT mice, and deletion of Gab2 remarkably reduced adipose tissue fibrosis remodeling caused by HFD (Fig. 4D, red arrow indicated). Moreover, picro-sirius staining of iWAT revealed that there were no obvious differences in fibrosis in iWAT from Gab2 KO and WT mice (Supplemental Fig. 3A).

Adipokines/chemokines secreted by adipose tissue can regulate metabolic homeostasis, while their secretion is affected by obesity^38^. Our results revealed that Gab2 ablation significantly elevated the *adiponectin* (*Adipoq*) mRNA expression (*P* < 0.001), and drastically reduced the mRNA expression of *leptin* (*P* < 0.01), *Tnf-α* (*P* < 0.05), *Il-1β* (*P* < 0.001) and *Il-6* (*P* < 0.001) (Fig. 4E). Furthermore, the expression of preceding molecules in blood circulation was detected in mice fed with HFD. Deletion of Gab2 increased the production of adiponectin, as well as suppressed the secretion of *leptin*, *Tnf-α*, *Il-1β*, and *Il-6* in HFD-fed mice (Fig. 4F–H). Our results indicated that Gab2 deficiency may contribute to reducing inflammation, which could be credited with the decreased macrophage infiltration in WAT of Gab2 KO mice.

### Deletion of Gab2 enhances the function of BAT in HFD-fed mice

**Fig. 5:**
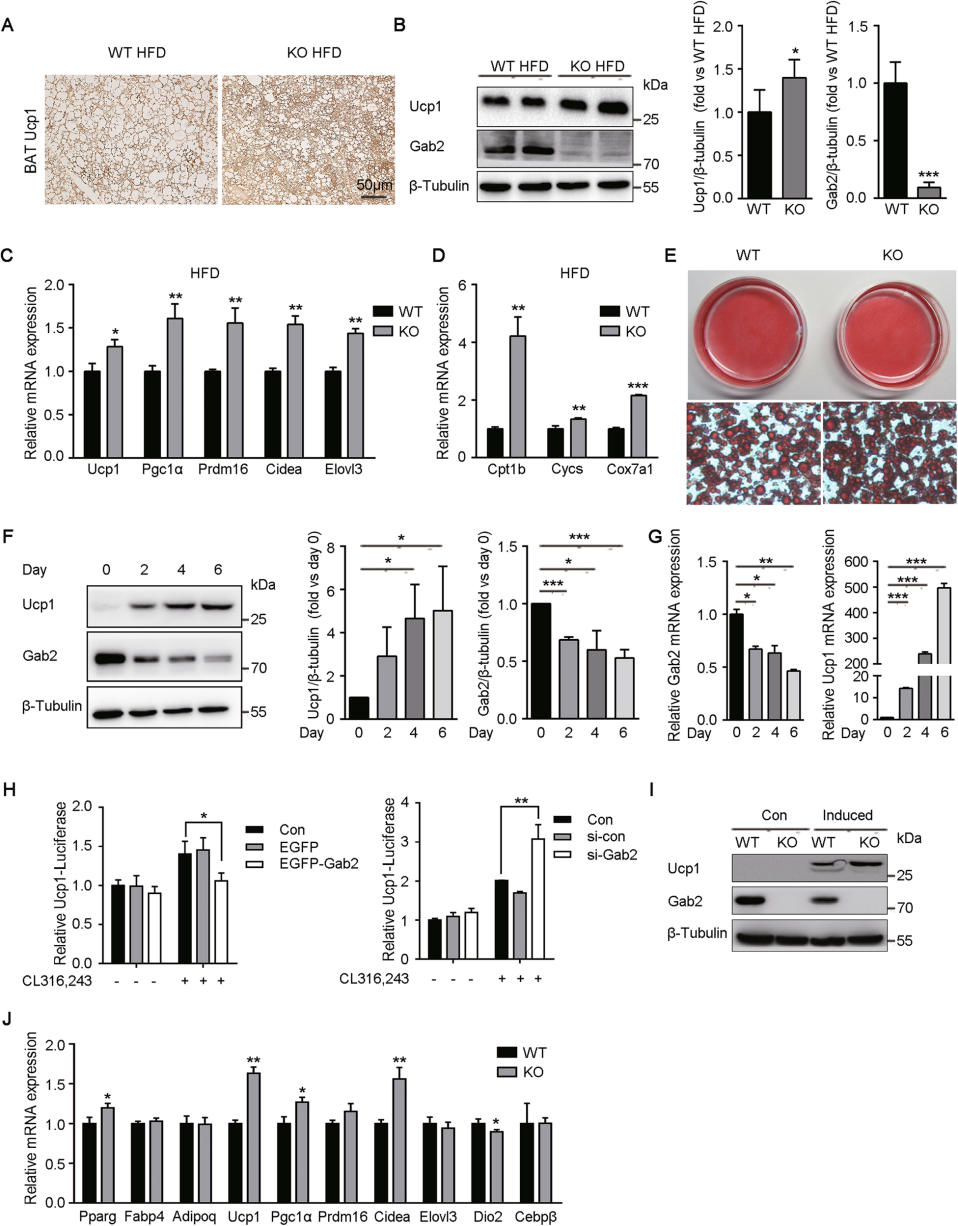
Gab2 deficiency increases the expression of thermogenesis-related genes in HFD-fed mice and differentiated brown adipocytes. **A** Representative IHC staining images of Ucp1 in BAT from Gab2 KO and WT mice fed with HFD diet for 12 weeks. The scale bar is 50 μm. **B** Left panel, representative blots of Ucp1 and Gab2 protein expressions in BAT from Gab2 KO and WT mice fed with HFD diet for 12 weeks detected by immunoblotting analysis. Middle and right panel, the relative grayscale values of Ucp1 (middle) and Gab2 (right) protein blots were normalized with the loading control protein β-Tubulin. **C**, **D** The mRNA level of thermogenesis-related genes (**C**) and mitochondrial function associated genes (**D**) in BAT from Gab2 KO and WT mice fed with HFD diet for 12 weeks detected by RT-qPCR. The results were normalized with *S18* mRNA levels. **E** Oil Red O staining of completely differentiated brown adipocytes (Day 6). **F** Left panel, representative blots of Ucp1 and Gab2 protein expressions during the differentiation of brown preadipocytes detected by immunoblotting analysis. Middle and right panel, the relative grayscale values of Ucp1 (middle) and Gab2 (right) protein blots were normalized with the loading control protein β-Tubulin. **G** The mRNA level of *Gab2* (left panel) and *Ucp1* (right panel) during the differentiation of brown preadipocytes detected by RT-qPCR. The results were normalized with *S18* mRNA levels. **H** Ucp1-luciferase reporter activity in response to Gab2 overexpression (left panel) or Gab2 knockdown (right panel) analyzed in immortalized brown preadipocytes by using the Dual-Luciferase Reporter Assay System (Promega). Con, immortalized brown preadipocytes only transfected with Ucp1-luciferase reporter and renilla; si-con, siRNA of control protein. **I** Representative blots of Ucp1 and Gab2 protein expressions in undifferentiated and differentiated brown adipocytes detected by immunoblotting analysis. β-Tubulin protein was measured as a loading control. Con, undifferentiated brown preadipocytes; Induced, differentiated brown adipocytes. **J** The mRNA level of common or brown fat-selective genes in differentiated brown adipocytes (Day 6) detected by RT-qPCR. The results were normalized with *S18* mRNA levels. The data are presented as mean ± SD from at least six mice in each group or three independent experiments. Statistical difference is indicated: *P < 0.05; **P < 0.01; ***P < 0.001.

Here, the function of BAT was evaluated by measuring the expression of Ucp1, a main regulator of energy dissipation in BAT. With IHC staining, the expression of Ucp1 was observed to be higher in BAT of Gab2 KO mice than that in WT mice after 12 weeks of HFD feeding (Fig. 5A, brown color) and quantitative analysis of Ucp1 protein confirmed that observation (Fig. 5B). Conversely, HFD induced Ucp1 to an equal extent in iWAT from Gab2 KO and WT mice (Supplemental Fig. 3B), which indicated that Gab2 participated in the induction of Ucp1 in BAT, but not in iWAT. In addition, Gab2 deficiency also significantly enhanced the mRNA expression of *Ucp1* and other thermogenic genes, including Ppar-γ coactivator-1α (*Pgc1α*), PRD1-BF1-RIZ1 homologous domain containing 16 (*Prdm16*), *Cidea*, and *Elovl3* (Fig. 5C). Furthermore, the genes associated with mitochondrial function and fatty acid oxidation, including carnitine palmitoyl transferase 1B (*Cpt1b*), cytochrome C *(Cycs*), and Cytochrome c oxidase subunit 7A1 (*Cox7a1*), were also highly expressed in BAT from Gab2 KO mice compared to that in WT mice fed with HFD (Fig. 5D).

We next examined whether Gab2 affects brown adipocyte differentiation by inducing primary brown preadipocytes differentiating in vitro. By evaluating with oil red O staining, both of primary brown preadipocytes with or without Gab2 have differentiated into mature adipocytes (Fig. 5E), in which the adipocytes markers, including *Ppar γ* (*Pparg*), *Fabp4* and *Adiponectin* (*Adipoq*), were expressed (Fig. 5J). Protein and mRNA of Ucp1 were gradually increased accompanied by the expression of Gab2 gradually reduced during the differentiation progress (Fig. 5F, G). To further explore the role of Gab2 in brown adipose tissue, an Ucp1-luciferase reporter plasmid was co-transfected with EGFP-Gab2 plasmid or si-Gab2 into immortalized brown preadipocytes, and then cells were stimulated with β3-adrenergic agonist CL316,243, an activator of Ucp1 transcription^39^. The results showed that overexpression of Gab2 suppressed the activity of the Ucp1 promoter (Fig. 5H, left panel), while knockdown of Gab2 significantly increased the transcription of Ucp1 (Fig. 5H, right panel), indicating that Gab2 may play a negative role in the differentiation of brown fat tissue.

At the end of differentiation progress, Ucp1 protein was dramatically increased fivefold and the expression of Gab2 was reduced by ~50% (Fig. 5F). Moreover, the mRNA expression of *Ucp1* was increased more than 490-fold and *Gab2* mRNA was reduced by ~56% during the differentiation process, which was consistent with protein expression changes (Fig. 5G). Further research revealed that Ucp1 protein level in differentiated Gab2 KO adipocytes was also higher than that in differentiated WT adipocytes (Fig. 5I). The expression of several brown fat-selective genes, including *Ucp1*, *Pgc-1α*, and *Cidea* was enhanced in differentiated adipocytes without Gab2 (Fig. 5J). These results confirmed the observation in mice and suggested that Gab2 may suppress the function of brown fat tissue.

### Gab2 regulates BAT function by PI3K-Akt-FoxO1 signaling pathways

**Fig. 6:**
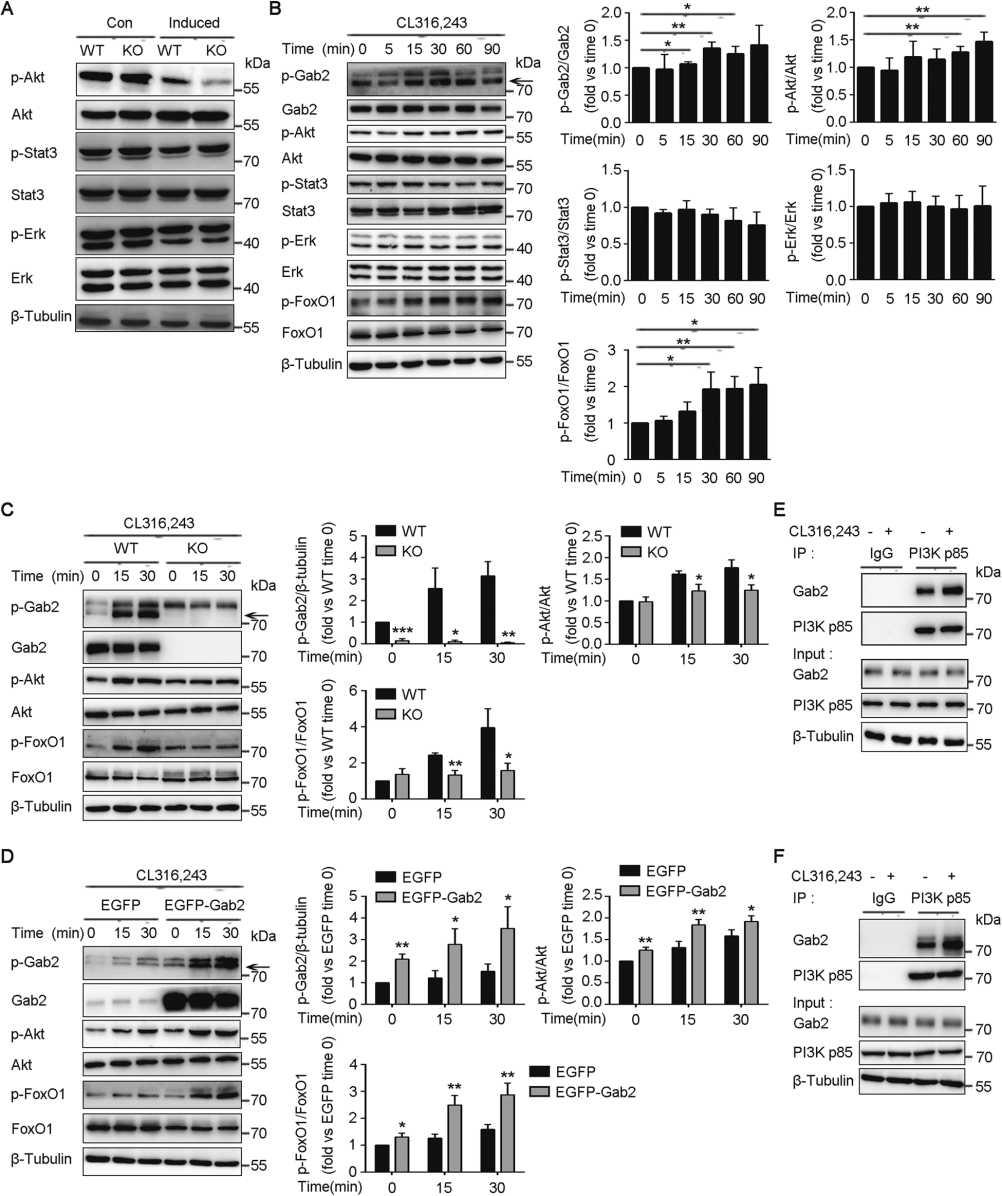
Gab2 regulates UCP1 expression by mediating PI3K p85-AKT-FoxO1 signaling pathways. **A** Representative blots of proteins in undifferentiated and differentiated brown adipocytes detected by immunoblotting analysis. β-Tubulin protein was measured as a loading control. Con, undifferentiated brown preadipocytes; Induced, differentiated brown adipocytes. **B** Representative blots of proteins in CL316,243-stimulated immortalized brown preadipocytes detected by immunoblotting analysis. The specific p-Gab2 band is indicated by the black arrows. The relative grayscale values of protein blots were normalized with the control protein. β-Tubulin protein was measured as a loading control. **C**, **D** Representative blots and relative grayscale values of Akt-FoxO1 signals in Gab2 deficiency primary brown preadipocytes (**C**) or in Gab2 overexpression immortalized brown preadipocytes (**D**) stimulated with CL316,243 detected by immunoblotting analysis. β-Tubulin protein was measured as a loading control. The specific p-Gab2 band is indicated by the black arrows. **E**, **F** The interaction between Gab2 and PI3K p85 in primary brown preadipocytes stimulated with CL316,243(5 μM) for 30 min (**E**) or in BAT of mice injected with CL316,243 (1 mg/kg/day) for 3 days (**F**) detected by immunoprecipitation. The data are presented as mean ± SD from three independent experiments. Statistical difference is indicated: *P < 0.05; **P < 0.01.

To explore the molecular mechanism of Gab2 regulating BAT function, the effect of Gab2 deficiency on its downstream signaling molecules Akt, Stat3, and Erk1/2 was investigated in undifferentiated primary brown preadipocytes and differentiated adipocytes. Gab2 deficiency did not affect its downstream signaling molecules in undifferentiated primary brown preadipocytes (Fig. 6A, left second bands). However, deletion of Gab2 reduced phosphorylation of Akt, while had no influence on the activation of Stat3, and Erk1/2 in the differentiated brown adipocytes (Fig. 6A, right first bands). Gab2 and its downstream signaling molecules were further detected in immortalized brown preadipocytes stimulated with CL316,243. The phosphorylation levels of Gab2(Tyr452) and Akt were gradually elevated in brown preadipocytes treated with CL316,243 for different times (Fig. 6B, top first and third panel). While the phosphorylation of Stat3 was decreased moderately and p-Erk was not discernibly changed in the stimulated cells (Fig. 6B, top fifth and seventh panel). It has been reported that the process of Akt regulating Ucp1 expression needs the assistance of transcription factor FoxO1^40,41^. Our result indicated that continuous stimulation with CL316,243 increased the phosphorylation level of FoxO1 in immortalized brown preadipocytes (Fig. 6B, top ninth panel). Further research revealed that the CL316,243-induced activation of Gab2, Akt, and FoxO1 in primary brown preadipocytes was suppressed by deletion of Gab2 (Fig. 6C). On the contrary, overexpression of Gab2 in immortalized brown preadipocytes enhanced the activation of Gab2, Akt, and FoxO1 induced by CL316,243 (Fig. 6D).

Gab2 could mediate the activation of Akt, which needs be phosphorylated on Tyr452 in Gab2 and combining with PI3K p85^42^. Hence, Primary brown preadipocytes isolated from BAT of WT mice were treated with CL316,243 for 30 min, then cells were collected to detect the combining capacity of PI3K p85 and Gab2. The results of the immunoprecipitation assay revealed that CL316,243 enhanced the binding of p85 with Gab2 (Fig. 6E). We also detected the combining capacity in brown adipose tissue of mice injected with CL316,243 for 3 days and found that CL316,243 also enhanced the binding of p85 with Gab2 in BAT, which is consistent with the result in primary brown preadipocytes (Fig. 6F).

These results suggest that Gab2 can negatively regulate the activation of the Ucp1 promoter in cells stimulated with CL316,243, and this regulation is possibly through Gab2 downstream PI3K (p85)-Akt-FoxO1 signaling pathways.

## Discussion

The present research revealed that deletion of Gab2 attenuated the weight gain caused by HFD, partly but importantly by attenuating adipose tissue inflammation and increasing the metabolic capacity of brown adipose tissue. The study reveals novel regulation of Gab2 on lipid homeostasis and adipocytes function, as well as provides a novel target for the treatment of obesity and associated complications.

Obesity is a disorder of systemic energy storage and dissipation characterized by the abnormal expansion of adipocytes and accumulation of triglyceride in cells^1^. Excess lipid accumulates in adipose tissue and liver and results in the hyperplasia and hypertrophy of adipocytes^6^. Our previous experiment discovered that Gab2 mediated the lipid metabolism in fatty liver disease and Gab2 deficiency prevented the lipid accumulation in the liver, indicating that Gab2 may be involved in obesity^31^. In our current research, Gab2 was found to be highly expressed in adipose tissue of HFD-fed mice and deletion of Gab2 suppressed the lipid accumulation in white adipose tissues, brown adipose tissue, and liver. The HFD-induced hyperplasia and hypertrophy of white adipocytes were remarkably diminished in iWAT by deletion of Gab2. Our observation demonstrated that Gab2 is a key regulator of obesity and suppression of Gab2 may improve the treatment of obesity.

The disorder of lipid and glucose metabolism is an important inducement for obesity^1^. We observed that deletion of Gab2 enhanced glucose tolerance and insulin sensitivity in both SD and HFD-fed mice. Insulin signal plays a critical role in the metabolic process and insulin resistance usually accompanies obesity, diabetes, and several other metabolic diseases^6^. the PI3K-Akt, Ras-Erk, Stat3, and other cytoplasmic signaling pathways associated with Gab2 can mediate the metabolic signals of insulin, IGF-1, and some inflammatory factors^23^. Liver-specific knockout of Gab1, another member of the Gab protein family, enhances insulin sensitivity and glucose tolerance^43^. Thus, Gab2 deficiency can directly improve glucose tolerance. In addition, many studies have shown that obesity is the main cause of metabolic syndrome, which includes elevated blood pressure, high blood sugar and other symptoms^1^. Overweight and obese men exhibit an increased risk of abnormal glucose regulation^1^. Furthermore, it has been proved that the cause of fatty liver disease can stimulate the expression of Gab2, which mediate the biosynthesis of lipids in the liver of mice^31^. Therefore, the lack of Gab2 not only directly affects glucose tolerance, but also has an indirect effect on glucose tolerance through weight loss. Our results suggested that Gab2 has the potential to regulate glucose homeostasis in obesity.

Inflammations in adipose tissue are associated with the development of obesity, insulin resistance, and other metabolic diseases^44^^,^^45^. Our research revealed that deletion of Gab2 reduced macrophage infiltration and fibrosis remodeling and affected the function of macrophages in adipose tissue. It is reported that Gab2 expresses in immune cells such as mast cells, T cells, and macrophages^24–27^ and exerts important biological functions. Deletion of Gab2 in animals makes the degranulation of mast cells defective^46^, as well as decreases the number of mast cells in different tissues^27^. Gab2 is essential for the CSF-1-dependent mononuclear phagocytes (MNPs) proliferation^25,26^. Moreover, deficiency of either Gab1 or Gab2 can meliorate BLM-induced pulmonary fibrosis by destroying M2 macrophage polarization^24^. In addition, obesity affects the secretion function of adipocytes, which is involved in immune cell recruitment and inflammatory responses^38^. Adiponectin participates in glucose regulation and fatty acid oxidation, as well as reduces inflammation within adipose tissue^47–49^. It is also reported that adiponectin promotes macrophage transformation to an anti-inflammation phenotype^50,51^. Leptin may affect macrophages infiltration to adipose tissue by regulating adhesion molecules in endothelial cells^35^. Here, the deletion of Gab2 may improve obesity by reducing recruitment of macrophages and secretion of inflammatory factors.

Recently, accumulating evidence shows that brown fat tissue (BAT) is a potential target for the prevention and treatment of obesity^9–11^. BAT is the major organ for non-shivering thermogenesis and diet-induced thermogenesis^52^, and involved in maintaining energy balance and resisting obesity by combusting glucose and triglycerides to generate heat. HFD induces the thermogenic program of different fat depots, including BAT and iWAT^53^. The enhancement of differentiation of BAT and activation of BAT was demonstrated to improve metabolic disorder and obesity. The heat production function of BAT depends on its specific gene Ucp1, which can dissipate the mitochondrial electrochemical gradient, uncouple respiration to generate heat^12^. Ucp1 is induced during the brown adipocyte differentiation and also thought as a marker of differentiation mature of brown adipocyte. Various factors, which can facilitate thermogenesis in BAT or regulate the brown adipocyte differentiation progress, may impact the quantity of Ucp1^54^. Our research in mice revealed that the induction of Ucp1 during brown preadipocyte differentiation in vitro was accompanied by the attenuation of Gab2 and deletion of Gab2 increased the expression of *Ucp1* in differentiated adipocytes. The results indicated that Gab2 may prevent the differentiation process of brown adipocytes. In both brown adipose tissue and primary adipocytes, deletion of Gab2 enhanced the expression of *Ucp1* and other thermogenic genes, suggesting that Gab2 may suppress the function of BAT.

The differentiation and function of BAT were guided by many factors and several signaling pathways. PI3K/Akt signaling is involved in the regulation of Ucp1 transcription, and PI3K inhibitor upregulated *Pgc1α* and *Ucp1* in brown adipocytes by recruiting FoxO1 to the gene promoter regions^40,41^. Stat3 can induce Ucp1 transcription and control the fate of brown adipocyte differentiation^55^. In addition, Jak2 plays an important role in regulating Ucp1 expression in BAT by regulating downstream Stat3 or Stat5 proteins^56^. Here, PI3K/Akt rather than Stat3 and Erk1/2 mediated the regulation of Gab2. PI3K/Akt pathway is involved in the Gab2 regulation in numerous tissues and mediates the signals of insulin, IGF-1, and some inflammatory factors^57^. In the liver, Gab2 is also employed the PI3K/Akt pathway to regulate lipid metabolism^31^. In summary, the PI3K/Akt pathway may be a key pathway in the regulation of Gab2 on lipid and glucose metabolism, inflammation, and the differentiation and function of adipose tissue in obesity.

Since we have used global Gab2 knock out animals for these studies, and multiple organs are involved in the maintenance of energy balance, such as liver, adipose tissue and muscle, it is possible that the changes in overall metabolism are a result of Gab2 multiple defects in more than one tissue. Gab2, as a critical integrator of various physiological pathways, is identified as a potential therapeutic target in many kinds of diseases. Gab2 is recruited by HFD to promote the development of obesity by regulating multiple pathways and the deletion of Gab2 resists HFD-induced obesity. Our current research exhibits the regulation of Gab2 on adipose tissue inflammation and brown adipose tissue function, which expands and emphasizes the therapeutic role of Gab2 and provides a novel target for the treatment of obesity and associated complications.

## Materials and methods

### Animals

Wild-type C57BL/6 mice were obtained from the Laboratory Animal Center of Xiamen University, China. Gab2 knockout mice have been described previously^58^. All mice were maintained in a SPF facility in a 12-hour light/dark cycle with unlimited access to food and water. In each experiment, male Gab2 KO mice and their WT littermates are randomly grouped into control or test group and each group contains 8 mice. All experimental procedures were approved by the Animal Welfare Committee of Research Organization (X200810), Xiamen University.

### High-fat diet-induced obesity models

Six-week-old male Gab2 KO mice and their WT littermates were fed with a standard diet (SD) or a high-fat diet (HFD) (60% fat, D12492, Research Diets) for 12 weeks. Bodyweight was weekly monitored. The total fat and lean mass were assessed with EchoMRI-100H (EchoMRI, USA). Food intake of different groups was monitored for at least one week before the end of the experiment. At the end of the experiment, mice were euthanized, tissues and blood samples were collected for further analysis.

### β3-adrenergic agonist treatments

In order to activate the thermogenesis function of BAT, male C57BL/6 mice at 3 months of age were intraperitoneally injected with 1 mg/kg/day CL316,243 (Sigma, St. Louis, MO, USA). Mice were euthanized after injection 3 times and adipose tissues were collected for further analysis.

### Blood biochemical analyses

The fasting plasma glucose concentration of mice fasted overnight was measured by using Roche ACCU-CHEK^TM^ glucometer. Blood samples were collected before the mice were sacrificed and serum was prepared by centrifuging at 3000 rpm for 10 min. Serum cytokines leptin (FineTest, P0191), Adiponectin (FineTest, EM001), TNF-α (Biotech, EM008), IL-1β (Biotech, EM001), IL-6 (Biotech, EM004) was evaluated by ELISA kit following standard protocol.

### Glucose and insulin tolerance (GTT & ITT)

Glucose tolerance test (GTT) and insulin tolerance test (ITT) were measured during the last week of HFD. For GTT, mice were fasted overnight with free access to water, followed by an oral administration of glucose (2 g/kg). The blood samples were collected from the caudal vein and the blood glucose concentration was measured by using Roche ACCU-CHEK^TM^ glucometer at 0, 15, 30, 60, 90, 120 min. For ITT, mice were fasted for 4 h followed by an intraperitoneal injection of human insulin (1 U/kg). Blood glucose concentration was measured at 0, 15, 30, 45, 60, 120 min and the results were expressed as the change of blood glucose relative to the basal level. The area under the curve (AUC) of GTT or ITT was calculated from the area between the base line and the indicated line using GraphPad software.

### Histological analyses

Mouse tissues were collected and fixed with 4% paraformaldehyde (PFA), dehydrated, and embedded in paraffin. Paraffin sections (5 μm) were prepared by Leica histological system. Hematoxylin and Eosin (H&E), immunohistochemistry (IHC), and Picro-Sirius staining were performed following standard protocol. Images were captured by using a Leica DM4 microscope and analyzed with Image-Pro software.

The size of adipocytes was analyzed with Image-Pro software from 3 to 4 low-power cross-sectional HE staining images of one mouse (3 mice per group). The density of crown-like structures (CLSs) was analyzed from IHC images of F4/80 (Abcam, ab111101) staining. Three to five low-power cross-sectional images of one mouse (three mice per group) were selected. The number of CLS and the total number of adipocytes were counted and the CLS density was presented as the ratio of CLS per 100 adipocytes^59^.

### Western blotting and coimmunoprecipitation

Protein was extracted from tissue with RIPA buffer (Tris-HCl (pH 7.4) 50 mM, NaCl 150 mM, NP-40 1%, Sodium deoxycholate 0.5%, SDS 0.1%, EDTA 1 mM). For immunoblotting assay, 40 μg protein samples were separated by SDS-PAGE, and detected with antibody against Gab2 (Cell Signaling, 3239), p-Gab2(Tyr452) (Cell signaling, 3881), Ucp1 (Abcam, ab10983), p-Akt(Ser473) (Cell Signaling, 4060), Akt(1/2/3) (Santa Cruz, 8312), p-Erk1/2(Thr202/Tyr204) (Cell Signaling, 9101), Erk (Cell Signaling, 9102), p-Stat3(Tyr705) (Cell Signaling, 9131), Stat3 (Cell Signaling, 9139), β-Tubulin (TransGen, HC101-01), PI3K p85 (Cell Signaling, 4257), p-FoXO1(Thr24)/3a(Thr32) (Cell Signaling, 9464), FoXO1 (Cell Signaling, 2880).

For coimmunoprecipitation analysis, adipose tissues or cell samples were lysed with lysis buffer (Tris-HCl (pH 7.5) 20 mM, NaCl 150 mM, TritonX-100 1%, glycerinum 5%, Sodium deoxycholate 1%, EDTA 5 mM, phosphatase inhibitor, protease inhibitor). After centrifuging (13,500 rpm, 4 °C, 15 min), lysates were applied for immunoprecipitation with antibodies coated protein G agarose beads at 4 °C overnight. Then the beads were washed with lysis buffer five times (30 s), and the immunoprecipitated results were analyzed by immunoblotting.

### Quantitative real-time PCR analyses

Total RNA was extracted from adipose tissues or cell samples with Tripure reagent (Thermofisher, USA) and purified by RNA mini columns (Qiagen, USA). The RNA was quantified and reverse transcribed to complementary DNA (cDNA) with a commercial kit for cDNA synthesis (TransGen, China). Quantitative real-time PCR was performed using SYBR Green Super Mix (Yeasen, China) on Bio Rad CFX96 real-time PCR system. Target primer sequences were represented in Table S1. The relative mRNA abundance of target gene was normalized to the internal control gene S18, and expressed as mean ± SD.

### Cell culture and cell treatments

Primary brown preadipocytes were obtained as described previously with a few changes^60^. BAT was dissected from newborn Gab2 KO and WT mice very carefully and washed with pre-cooling PBS buffer for three times. Then the BAT was minced into 1 mm^3^ fragment, digested with collagenase A (Roche, 1.5 mg/mL) in isolation buffer (123 mM NaCl, 5 mM KCl, 1.3 mM CaCl2, 5 mM glucose, 100 mM HEPES, and 4% BSA) for 30 min at 37 °C. Well dispersed tissue was centrifuged at 200 x *g* for 5 min after filtering through 100 μM cell strainer. The obtained cells were washed and centrifuged one more time before resuspended in culture medium (high-glucose DMEM medium (Hyclone), 20% FBS, 20 mM HEPES). Cells were plated in 6-well plates and grew in a humidified atmosphere of 5% CO_2_ at 37 °C for further research.

The immortalized brown preadipocytes were a generous gift from Professor Wanzhu Jin (Institute of Zoology, Chinese Academy of Sciences) and were cultured in high-glucose DMEM medium containing 20% FBS. The isolated brown preadipocytes were immortalized by retroviral expression of the SV40 Large T antigen as previously described^55^.

For brown preadipocytes differentiation, when primary brown preadipocytes reached to 100% confluence for 2 days (Day 0), the cells were treated by induction medium high-glucose DMEM medium (Hyclone), 10% FBS, 20 nM insulin, 1 nM triiodothyronine (T3), 0.5 mM isobutylmethylxanthine (IBMX), 125 nM indomethacin, 1 μM dexamethasone) for 48 h. Then the cells were cultured in differentiation medium (high-glucose Dulbecco’s modified Eagle medium (DMEM) medium, 10% FBS, 20 nM insulin, 1 nM T3) for 4 days. The medium was changed every other day.

Gab2 expression plasmid was generated by cloning Gab2 cDNA fragment into plasmid pIRES2-EGFP, which contains a neomycin-resistance cassette (Neo^r^). In order to obtain Gab2 overexpression in cells, Gab2 expression plasmid (Gab2-EGFP) was transfected into immortalized brown preadipocytes for 24 h, after which CL316.243 (5 μM final concentration) was administrated to treat cells for further research.

Gab2 knockdown was mediated by siRNA. Si-Gab2 was purchased from Guangzhou RiboBio Co., Ltd. China and the sequence is GGAGTGCCAGCTTCTCTCA.

To stimulate the expression of thermogenesis genes, cells with 90% confluence were treated with CL316.243 (5 μM final concentration) for a predefined time.

### Luciferase reporter assay

The −4.16 kb Ucp1-luciferase reporter gene (a kind gift from Professor Wanzhu Jin in Institute of Zoology, Chinese Academy of Sciences) was performed in immortalized brown preadipocytes plated in 12-well plates. Briefly, Ucp1 reporter gene, Renilla and Gab2 expression plasmid (Gab2-EGFP) were co-transfected into cells using Lipofectamine 2000 (Invitrogen). After transfection for 24 h, cells were harvested and extracted to measure the reporter gene activity by Promega Dual-Luciferase reporter system. CL316,243 (5 μM) was added for 6 h, respectively, prior to harvesting cells. To transiently knock down Gab2 in immortalized brown preadipocytes, si-Gab2 was transfected into cells 12 h prior to the transfection of Ucp1 reporter gene. The following experiment was consistent with the process described above. The reporter gene results were normalized to Renilla luciferase activity.

### Oil red O staining

The fully differentiated brown adipocytes described previously were applied for Oil Red O staining. After discarding the medium, PBS buffer washed the cell for once, and 10% formalin was used to fix cells for 30 min. Cells were washed again and strained with freshly prepared Oil Red O (0-0625, Sigma) work solution for 1 h. Then the plates were washed several times and the accumulation of lipid content in adipocytes was evaluated by microscope.

### Statistical analysis

Difference between two groups were applied for unpaired two-tailed Student’s *t*-tests. Data involved more than two groups were analyzed by an unbalanced two-way analysis of variance (ANOVA) by using GraphPad Prism 7 software. All values are expressed as mean ± SD. For all tests, *P < 0.05, **P < 0.01, ***P < 0.001 were considered statistically significant.

## Supplementary information

Supplemental figure 1

Supplemental figure 2

Supplemental figure 3

Supplemental figure legends

Supplemental table 1
