# Supplementary figures and images for: Gab2 deficiency suppresses high-fat diet-induced obesity by reducing adipose tissue inflammation and increasing brown adipose function in mice
Source: Cell Death Dis. 2021 Feb 26;12(2):212. doi: 10.1038/s41419-021-03519-9 (PMC7910586; doi:10.1038/s41419-021-03519-9)

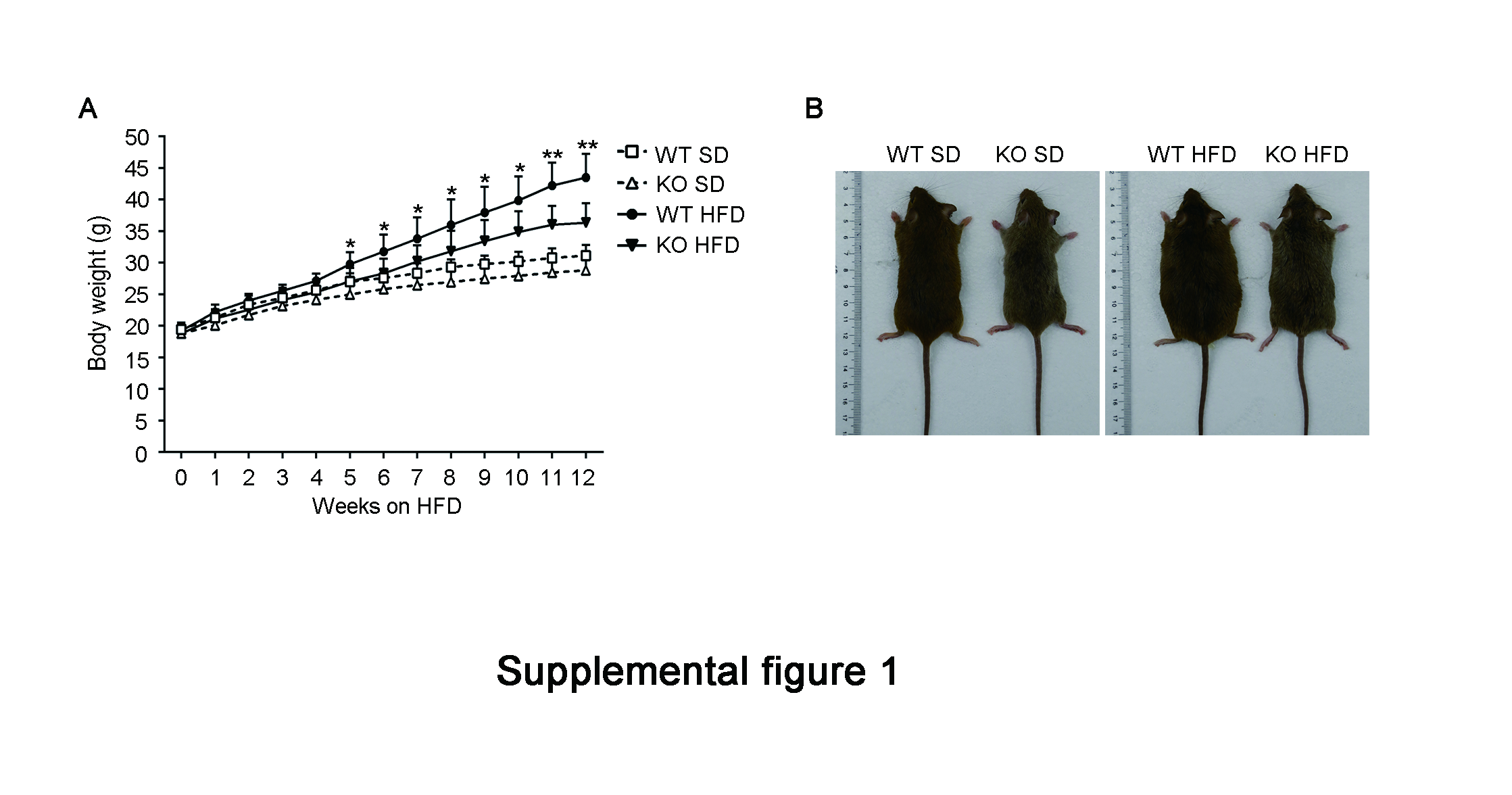

Supplement: Supplementary file 1 — Supplemental figure 1 [file 41419_2021_3519_MOESM1_ESM.tif]

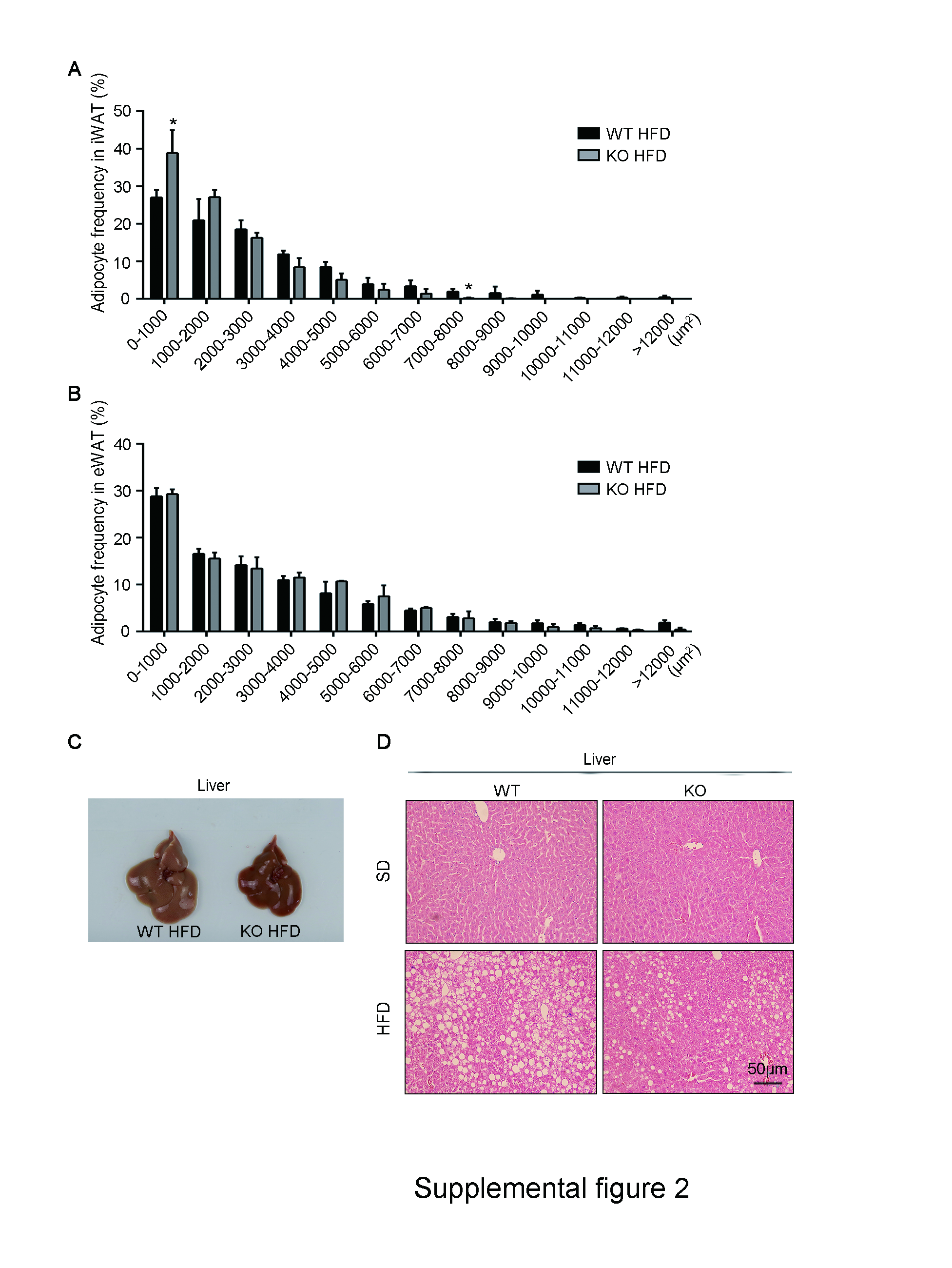

Supplement: Supplementary file 2 — Supplemental figure 2 [file 41419_2021_3519_MOESM2_ESM.tif]

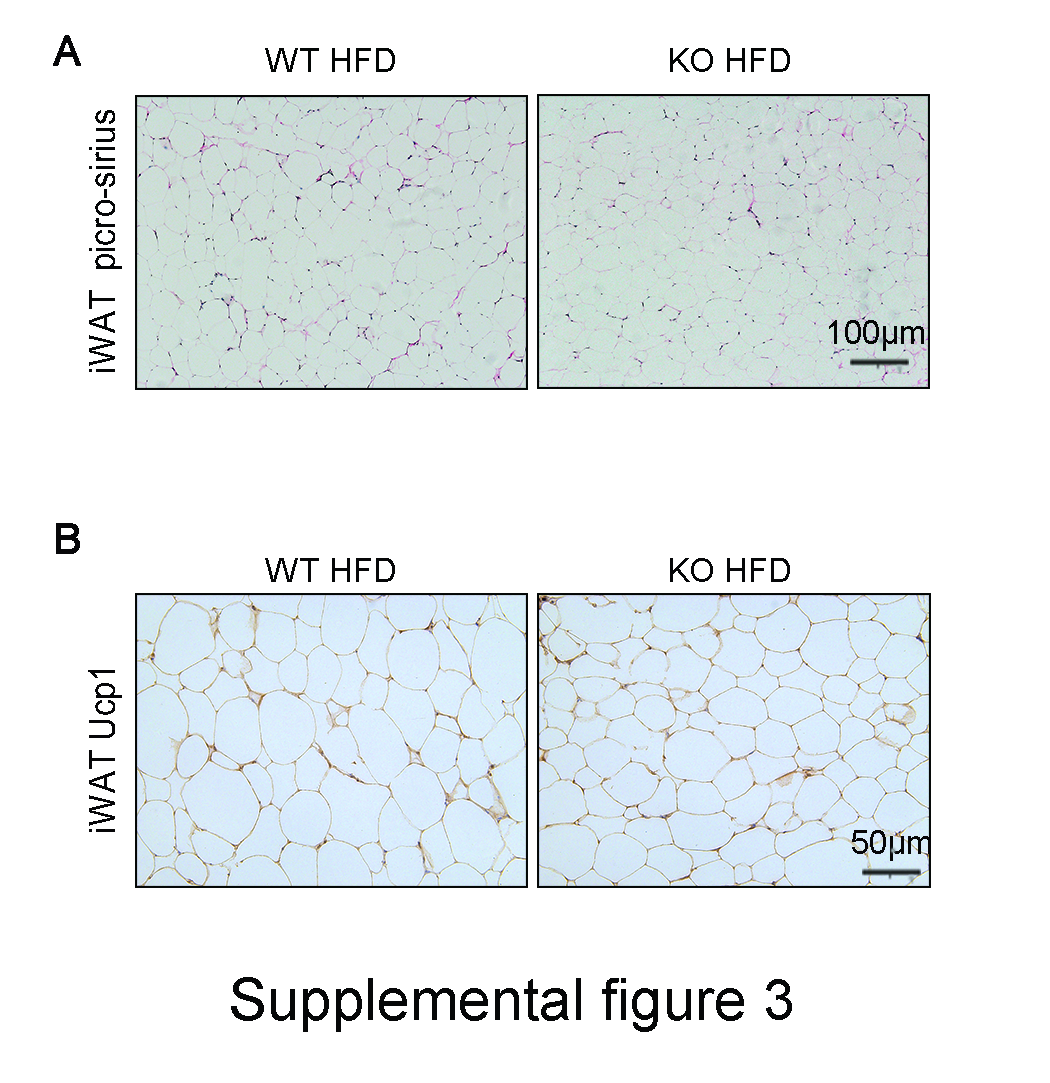

Supplement: Supplementary file 3 — Supplemental figure 3 [file 41419_2021_3519_MOESM3_ESM.tif]
