# Supplementary material for: Gab2 deficiency suppresses high-fat diet-induced obesity by reducing adipose tissue inflammation and increasing brown adipose function in mice
Source: Cell Death Dis. 2021 Feb 26;12(2):212. doi: 10.1038/s41419-021-03519-9 (PMC7910586; doi:10.1038/s41419-021-03519-9)
Supplement: Supplementary file 4 — Supplemental figure legends [file 41419_2021_3519_MOESM4_ESM.docx]

**Supplemental figure legends**

**Supplemental Figure 1. Deletion of Gab2 protects against HFD-induced obesity. (A)** Bodyweight gain curve of Gab2 KO and WT mice fed with SD and HFD diet for 12 weeks. **(B)** Representative photographs of mice at 18 weeks of age. The data are presented as mean ± SD from at least 6 mice in each group. Statistical difference is indicated: **P* < 0.05; ***P* < 0.01.

**Supplemental Figure 2. (A, B)** Adipocyte size distribution of iWAT (A) and eWAT (B) from Gab2 KO and WT mice fed with HFD diet for 12 weeks. **(C, D)** Representative image (C) and hematoxylin and eosin (H&E) staining (D) of liver from Gab2 KO and WT mice fed with SD and HFD diet for 12 weeks, scale bar is 50 μm. The data are presented as mean ± SD. Statistical difference is indicated: **P* < 0.05;

**Supplemental Figure 3. (A)** Representative picro-sirius staining images of collagen fibrils in iWAT from Gab2 KO and WT mice fed with HFD diet for 12 weeks, scale bar is 100 μm. **(B)** Representative IHC staining images of Ucp1 in iWAT from Gab2 KO and WT mice fed with HFD diet for 12 weeks. The scale bar is 50 μm.
