# Supplementary material for: Gab2 deficiency suppresses high-fat diet-induced obesity by reducing adipose tissue inflammation and increasing brown adipose function in mice
Source: Cell Death Dis. 2021 Feb 26;12(2):212. doi: 10.1038/s41419-021-03519-9 (PMC7910586; doi:10.1038/s41419-021-03519-9)
Supplement: Supplementary file 5 — Supplemental table 1 [file 41419_2021_3519_MOESM5_ESM.docx]

| Table S1 RT-qPCR Target Gene Primer Sequence | |
| --- | --- |
| Gene | Primer Sequence (5' to 3') |
| S18-F | TCCAGCACATTTTGCGAGTA |
| S18-R | CAGTGATGGCGAAGGCTATT |
| adiponectin-F | GCACTGGCAAGTTCTACTGCAA |
| adiponectin-R | GTAGGTGAAGAGAACGGCCTTGT |
| C/EBPβ-F | GACTGACGCAACACACGTGTAA |
| C/EBPβ-R | ATCAACAACCCCGCAGGAA |
| Cidea-F | TGCTCTTCTGTATCGCCCAGT |
| Cidea-R | GCCGTGTTAAGGAATCTGCTG |
| Cox7a1-F | CGAAGAGGGGAGGTGACTC |
| Cox7a1-R | AGCCTGGGAGACCCGTAG |
| Cpt1b-F | CAAGTCATGGTGGGCAACTA |
| Cpt1b-R | GCTGCTTGCACATTTGTGTT |
| Cytochrome C1-F | GCTACCCATGGTCTCATCGT |
| Cytochrome C1-R | CATCATCATTAGGGCCATCC |
| DIO2- F | CTGCGCTGTGTCTGGAAC |
| DIO2- R | GGAGCATCTTCACCCAGTTT |
| Elovl3-F | TCCGCGTTCTCATGTAGGTCT |
| Elovl3-R | GGACCTGATGCAACCCTATGA |
| Fabp4-F | ACACCGAGATTTCCTTCAAACTG |
| Fabp4-R | CCATCTAGGGTTATGATGCTCTTCA |
| F4/80-F | TTTCCTCGCCTGCTTCTTC |
| F4/80 -R | CCCCGTCTCTGTATTCAAC |
| Gab2-F | TCTGAGACTGATAACGAGGAT |
| Gab2-R | GATGGAGTCGGCTGTTG |
| IL-1β-F | AGATGAAGGGCTGCTTCCAAA |
| IL-1β-R | GGAAGGTCCACGGGAAAGAC |
| IL-6-F | CTGCAAGAGACTTCCATCCAG |
| IL-6-R | AGTGGTATAGACAGGTCTGTTGG |
| Leptin -F | GTGGCTTTGGTCCTATCTGTC |
| Leptin -R | CGTGTGTGAAATGTCATTGATCC |
| MCP- 1-F | CCACTCACCTGCTGCTACTCA T |
| MCP-1- R | TGGTGATCCTCTTGTAGCTCTCC |
| PGC-1α-F | CCCTGCCATTGTTAAGACC |
| PGC-1α-R | TGCTGCTGTTCCTGTTTTC |
| PPARg-F | GTGCCAGTTTCGATCCGTAGA |
| PPARg-R | GGCCAGCATCGTGTAGATGA |
| PRDM16-F | CAGCACGGTGAAGCCATTC |
| PRDM16-R | CACCTCTGTATCCGTCAGCA |
| TNF α-F | CAGGCGGTGCCTATGTCTC |
| TNF α-R | CGATCACCCCGAAGTTCAGTAG |
| UCP1-F | GGCAAAAACAGAAGGATTGC |
| UCP1-R | TAAGCCGGCTGAGATCTTGT |
